# Whole-Genome Characterization of a G3P[59] Rotavirus A Strain from a Diarrheic Donkey Foal Reveals an Unusual Genomic Background

**DOI:** 10.3390/ani16213511

**Published:** 2026-11-06

**Authors:** Qiyuan Liu, Kaikai Yu, Zaiyong Han, Rui Liang, Liying Guo, Mengling Ming, Yiming Xu, Wenhao Chen, Min Wang, Xiao Liang, Jelle Matthijnssens, Takeshi Haga, Jianbao Dong

**Affiliations:** 1 College of Veterinary Medicine, Qingdao Agricultural University, Qingdao 266109, China; liuqiyuan@stu.qau.edu.cn (Q.L.); guoliying@stu.qau.edu.cn (L.G.); xuyiming111@stu.qau.edu.cn (Y.X.); chenwenhao@stu.qau.edu.cn (W.C.);; 2 College of Animal Science and Technology, Qingdao Agricultural University, Qingdao 266109, China; yukaikai@stu.qau.edu.cn (K.Y.); liangrui@stu.qau.edu.cn (R.L.); mingmengling@stu.qau.edu.cn (M.M.); min@qau.edu.cn (M.W.); 3 Laboratory of Clinical and Epidemiological Virology, Department of Microbiology, Immunology and Transplantation, Rega Institute, KU Leuven, 3000 Leuven, Belgium; jelle.matthijnssens@kuleuven.be; 4 Department of Veterinary Medical Science, Graduate School of Agriculture and Life Sciences, The University of Tokyo, Tokyo 113-8657, Japan; ahaga@g.ecc.u-tokyo.ac.jp

**Keywords:** rotavirus A, donkey, G3P[59], P[59], whole-genome sequencing, reassortment, phylogeny, cross-species evolution

## Abstract

Rotavirus A (RVA) is an important enteric pathogen of neonatal animals, whereas the genomic diversity of donkey-origin RVA remains incompletely defined. In 2025, an RVA strain was recovered from a diarrheic donkey foal in Shandong Province, China, and designated RVA/Donkey-wt/CHN/SD2503/2025/G3P[59] (SD2503). Viral RNA was subjected to Illumina NovaSeq paired-end sequencing, de novo assembly, genotype assignment, sequence comparison, and segment-specific maximum-likelihood phylogenetic analysis. Sequences corresponding to all 11 genome segments were recovered, yielding the constellation G3-P[59]-I3-R3-C3-M3-A9-N3-T3-E3-H6. The *VP4* gene was highly divergent: the closest BLASTn match was the P[24] rhesus strain TUCH, with 79.58% nucleotide identity, below the 80% threshold used to assign *VP4* sequences to the same P genotype. The *VP4* sequence was subsequently confirmed by the Rotavirus Classification Working Group as P[59]. Other segments showed discordant relationships. *VP7* clustered with rabbit strain rab1404 with strong bootstrap support, whereas several internal genes grouped within genotype backgrounds represented by simian, rabbit, pangolin, equine, and other mammalian RVAs. The topological incongruence across segments, together with the unusual genotype constellation, suggests possible historical reassortment involving cross-species-associated RVA lineages, although the specific reassortment events remain unresolved. SD2503 provides an 11-segment genomic framework for an independently identified donkey G3P[59] RVA and supports broader genomic surveillance of RVA in equids.

## Simple Summary

Rotavirus A is a significant cause of diarrhea in young animals, but viruses circulating in donkeys remain poorly characterized. We investigated a rotavirus recovered from a diarrheic donkey foal in Shandong Province, China, in 2025. Sequences corresponding to all 11 rotavirus genome segments were recovered from SD2503, yielding the genotype constellation G3-P[59]-I3-R3-C3-M3-A9-N3-T3-E3-H6. Its *VP4* gene was especially divergent from previously recognized P genotypes and was confirmed by the Rotavirus Classification Working Group as P[59]. Phylogenetic analyses of the 11 segments did not place the virus consistently with a single host-associated lineage. Instead, different genes were related to rotaviruses previously detected in rabbits, horses, simians, pangolins, donkeys, and other mammals. This mosaic pattern is consistent with possible historical reassortment, a process in which segmented viruses exchange genome segments during mixed infection, although the specific events cannot be determined from the available data. The findings expand the known diversity of donkey rotaviruses and show why whole-genome surveillance is important for identifying unusual viruses that cannot be adequately understood from VP7 and VP4 typing alone.

## 1. Introduction

Rotavirus A (RVA) is a segmented double-stranded RNA virus and a major cause of acute gastroenteritis in the young of humans and many mammalian species [1,2,3]. Its 11 genome segments encode six structural proteins (VP1–VP4, VP6 and VP7) and five or six nonstructural proteins (NSP1–NSP5/6). The segmented genome provides repeated opportunities for reassortment when genetically distinct RVAs coinfect the same cell, and this process is a major contributor to rotavirus diversity and host-associated evolution [2,3,4,5,6].

A whole-genome nomenclature is therefore used for RVA. Genotypes are assigned in the order Gx-P[x]-Ix-Rx-Cx-Mx-Ax-Nx-Tx-Ex-Hx, corresponding to VP7, VP4, VP6, VP1, VP2, VP3 and NSP1–NSP5, respectively [4,5]. This approach is particularly informative for unusual animal viruses because identical G/P combinations can occur on different internal-gene backgrounds. For *VP4*, an 80% nucleotide identity cutoff is used for assignment to the same P genotype [4,5].

Equine RVA is an important cause of diarrhea in foals, and G3P[12] and G14P[12] account for most well-characterized equine strains worldwide [7,8,9,10,11,12]. Nevertheless, atypical equine RVAs provide evidence that equids can acquire viruses or genome segments associated with other mammalian hosts. The Argentine strain E3198 possessed the constellation G3-P[3]-I3-R3-C3-M3-A9-N3-T3-E3-H6 and was related to simian and feline/canine-like RVAs [10]. Indian equine G3P[3] strains have also been shown to possess bat-associated genomic backgrounds [13].

Information on RVA infection in donkeys is limited. A severe enteritis outbreak in donkey foals in Shandong Province in 2019 was associated with RVA/Donkey-wt/CHN/Don01/2019/G3P[12]; its *VP7* gene was atypical and related to bat-associated G3 viruses, whereas *VP4* was equine-like P[12] [14]. A later Chinese donkey strain, HB01/China/2021/G3P[12], was also placed near atypical G3 lineages in *VP7* analyses [15]. In 2025, another donkey-associated RVA strain, HB2503, was identified in Hebei Province, China, and subsequently described in a study focused on the development and validation of an antigen-capture ELISA for donkey-origin RVA [16].

Here, we report the whole-genome characterization of SD2503, a G3P[59] rotavirus A strain independently isolated from a diarrheic donkey foal in Shandong Province, China, in 2025. To the best of our knowledge, this study represents the first publicly reported eleven-segment genomic characterization and comprehensive phylogenetic analysis of a donkey-associated G3P[59] RVA strain. By determining its complete genotype constellation and examining the evolutionary relationships of individual genome segments, our findings provide a genomic reference for investigating the diversity and evolutionary history of this unusual P genotype in equids. From an epidemiological perspective, the identification of this unusual genotype highlights the need for broader surveillance to determine the prevalence, host distribution, and clinical significance of P[59] RVA strains in equids.

## 2. Materials and Methods

### 2.1. Sample Origin and Virus Isolation

In 2025, a fecal sample was collected after natural defecation from a 2-day-old donkey foal with diarrhea in Shandong Province, China. The foal appeared clinically healthy at birth, was able to stand approximately 50 min after birth, and began suckling colostrum approximately 55 min after birth. No apparent clinical abnormalities were observed during the early neonatal period. However, watery diarrhea developed approximately 40 h after birth. The fecal sample was collected non-invasively, without direct contact with or handling of the animal. The fecal material was suspended in sterile phosphate-buffered saline containing antibiotics and clarified by centrifugation. The supernatant was passed through a 0.22 μm membrane. The filtrate was mixed with an equal volume of Dulbecco’s modified Eagle medium (DMEM) containing 10 μg/mL crystalline trypsin and incubated at 37 °C for 60 min. Activated material was inoculated onto confluent Madin–Darby bovine kidney (MDBK) cell monolayers and allowed to adsorb for 1 h at 37 °C. Monolayers were then washed three times with serum-free DMEM and maintained in trypsin-supplemented DMEM. Cultures were examined daily for cytopathic effects (CPE). Blind passages were performed at 2–3-day intervals following three freeze–thaw cycles. CPE was first observed during the fourth passage. Culture material collected from each passage was tested for RVA RNA by RT-PCR targeting the *VP6* gene [17], with positive results obtained throughout the passage series. The identity of the recovered virus was further supported by transmission electron microscopy.

RVA RNA was detected using a one-step reverse transcription-polymerase chain reaction (RT-PCR) assay targeting the *VP6* gene, as previously described [17]. The primer pair consisted of BRVF (5′-ATGGGTACGATGTGGCTCAA-3′) and BRVR (5′-ACCGCTGGTGTCATGTTTGG-3′), yielding an expected amplicon of 383 bp. RT-PCR was performed using the TaKaRa One Step RNA PCR Kit (AMV). The reaction conditions were as follows: reverse transcription at 50 °C for 30 min; initial denaturation at 94 °C for 5 min; 35 cycles of denaturation at 94 °C for 50 s, annealing at 55 °C for 50 s, and extension at 72 °C for 1 min; followed by a final extension at 72 °C for 10 min. The assay was used to detect RVA RNA in the original fecal sample and in culture material collected during serial passage in MDBK cells.

### 2.2. Electron Microscopy

Virus-containing material was examined by transmission electron microscopy (TEM) using a negative-staining procedure. Briefly, 20 μL of the virus suspension was applied to a 200-mesh carbon-coated copper grid and allowed to adsorb for 3–5 min. Excess liquid was removed with filter paper, after which the grid was negatively stained with 2% phosphotungstic acid for 1–2 min. Excess stain was removed with filter paper, and the grid was allowed to dry at room temperature. The prepared grids were examined using an HT7800 transmission electron microscope (Hitachi High-Tech Corporation, Tokyo, Japan), and representative viral particles were imaged.

### 2.3. RNA Library Preparation and Whole-Genome Sequencing

Virus-containing culture material obtained following propagation of SD2503 in MDBK cells, rather than the original fecal specimen, was used for whole-genome sequencing. Viral RNA was extracted using TRIzol reagent and purified by the sequencing service provider, Shanghai Tanpu Biotechnology Co., Ltd. (Shanghai, China). RNA quality was assessed by concentration measurement and conventional gel electrophoresis. According to the provider’s protocol, no separate double-stranded RNA (dsRNA) denaturation step was performed. The extracted RNA was processed for library preparation, including reverse transcription using random hexamer primers and second-strand cDNA synthesis. Sequencing libraries were prepared using the Illumina Nextera XT library preparation kit and sequenced on an Illumina NovaSeq 6000 platform with 150 bp paired-end reads.

The sequencing run generated 12,805,186 reads before filtering and 12,680,940 reads after quality control, corresponding to 1,815,186,829 retained bases. The post-filter Q20 and Q30 rates were 97.20% and 93.51%, respectively, and the mean retained read length was 143 bp.

### 2.4. Read Processing, Assembly and Genome Annotation

Raw sequencing reads were quality-filtered and adapter-trimmed using fastp v0.20.0 [18]. Ribosomal RNA, host-derived, and bacterial reads were removed using the sequencing provider’s BBMap-based filtering workflow (v38.51). De novo assembly was performed using SPAdes v3.14.1 and SOAPdenovo v2.04 [19]. Virus-associated contigs were identified by nucleotide sequence comparison against reference databases. Coding regions were annotated using Prokka v1.14.5 and Prodigal v0.9, followed by manual comparison with RVA reference sequences [20,21]. The overall metagenomic assembly contained non-RVA contigs and was therefore not interpreted as the length of a single viral genome. Eleven contigs corresponding to VP1–VP4, VP6, VP7, and NSP1–NSP5 were retained for SD2503 genome analysis. The sequencing provider’s archived documentation did not include the complete command-line settings or project-specific parameters used for these preprocessing, assembly, and annotation steps; therefore, these parameters could not be independently verified.

To further evaluate sequencing support for the 11 assembled RVA genome segments, the paired-end FASTQ reads supplied for the SD2503 sequencing project were mapped against the assembled segment sequences using BWA-MEM (BWA v0.7.19-r1273) [22] with four alignment threads. The resulting alignments were sorted and indexed using SAMtools v1.22.1 [23]. Alignment statistics were obtained using the samtools flagstat and samtools idxstats commands, and per-base sequencing depths, including zero-coverage positions, were calculated using samtools depth -aa. For each segment, the mean, median, minimum, and maximum sequencing depths and the breadths of coverage at ≥1×, ≥10×, and ≥100× were calculated. Coverage breadth was defined as the percentage of positions within each assembled reference sequence meeting the specified sequencing-depth threshold. These analyses assessed read support for the assembled sequences but did not independently verify the complete 5′ and 3′ genomic termini.

### 2.5. Genotype Assignment and Sequence Comparison

Segment genotypes were assigned according to the RCWG whole-genome classification framework [4,5]. BLASTn searches were used to identify the closest available nucleotide matches. The *VP4* sequence showed less than 80% nucleotide identity to the closest previously recognized P genotype in the available BLASTn analysis. Following earlier scientific communication, the *VP4* sequence was formally submitted to the Rotavirus Classification Working Group (RCWG) for genotype evaluation on 4 October 2026. Its assignment to genotype P[59] was subsequently confirmed in an official letter dated 29 October 2026, signed by the Chair of the RCWG on behalf of its members. The nucleotide sequences of the 11 SD2503 genome segments were deposited in GenBank under accession numbers QB141057–QB141067.

### 2.6. Phylogenetic Analysis

Reference sequences used for phylogenetic analysis were retrieved from the NCBI GenBank database and selected based on nucleotide sequence similarity, genotype assignment, host origin, and phylogenetic relevance. The datasets included closely related database matches, relevant equine and donkey strains, and representative RVA lineages from other mammalian hosts. These reference datasets were intended to provide representative phylogenetic comparisons rather than an exhaustive assessment of global RVA diversity. The strain designations, GenBank accession numbers, host species, and available genotype information of the reference sequences are provided in Supplementary Table S1.

Nucleotide sequences were aligned using MAFFT v7 [24]. Maximum-likelihood phylogenetic analyses were performed in MEGA12 [25]. For each segment, the best-fitting nucleotide substitution model was identified using the maximum-likelihood model-selection procedure and the Bayesian information criterion. Trees were reconstructed using the selected model with 1000 standard bootstrap replicates. All three codon positions and noncoding positions present in the nucleotide alignments were included in the phylogenetic analyses. Gaps and missing sequence information were handled using the “Use all sites” option in MEGA12, under which alignment columns are retained rather than removed by complete- or partial-deletion filtering, while gaps and missing characters do not contribute nucleotide substitution information. No additional alignment-trimming or site-exclusion procedure was applied. Bootstrap percentages ≥70 are displayed. SD2503 is indicated by a black diamond (◆). Because different reference sets were required for different segments, branch placement was interpreted segment by segment rather than as evidence of direct ancestry. For Supplementary Table S3, nucleotide sequence identities between SD2503 and selected phylogenetically related reference strains were calculated using global pairwise alignments. Identity percentages were determined from positions at which both sequences contained an unambiguous nucleotide (A, C, G, or T), excluding alignment gaps and ambiguous characters. These pairwise comparisons were interpreted separately from the closest-match BLASTn results.

### 2.7. Use of Generative Artificial Intelligence

During manuscript preparation, ChatGPT (OpenAI; GPT-5.6 Sol and GPT-6 models) was used to assist with language editing, organization of author-provided information, and formatting. The tool was not used to generate experimental data or figures or to perform sequence or phylogenetic analyses. All scientific content and interpretations were reviewed and verified by the authors.

## 3. Results

### 3.1. Isolation and Morphological Identification of SD2503

**Figure 1:**
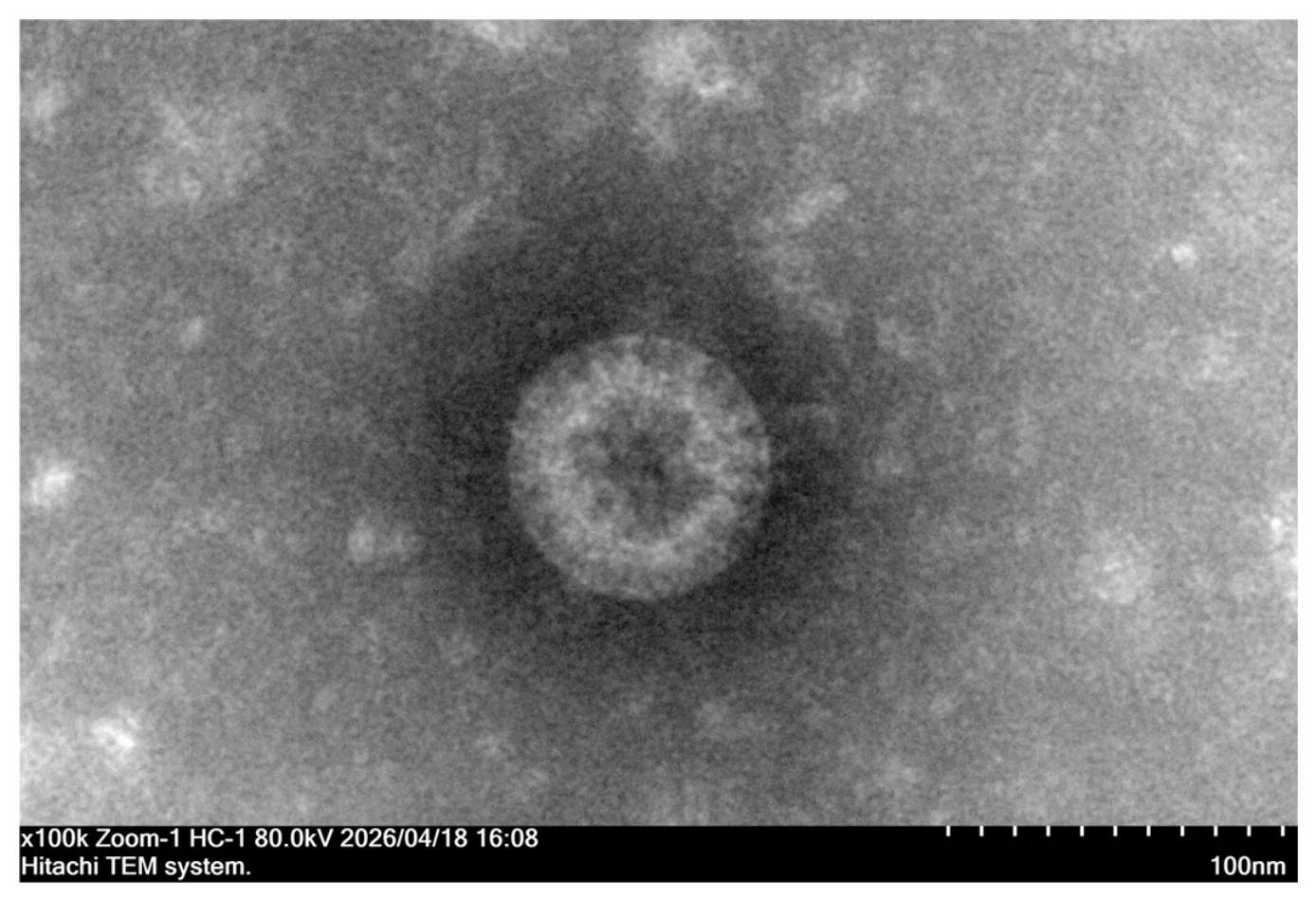
Representative transmission electron micrograph of the SD2503 virus preparation. A rotavirus-like particle is visible in the field. Scale bar, 100 nm.

Following inoculation of processed fecal material onto MDBK cells and serial blind passage, cytopathic effects were first observed at passage 4. The affected cells exhibited cytoplasmic vacuolation, rounding, and detachment from the cell monolayer. RVA RNA was detected by *VP6*-targeted RT-PCR in culture material from each passage. Transmission electron microscopy revealed a rotavirus-like particle exhibiting the characteristic wheel-like morphology (Figure 1). Together, these observations were consistent with the recovery and propagation of RVA in MDBK cells. The strain was designated RVA/Donkey-wt/CHN/SD2503/2025/G3P[59] (SD2503).

### 3.2. Sequencing Output and Genome Organization

The sequencing dataset retained 12.68 million reads after quality filtering, with Q20 and Q30 rates of 97.20% and 93.51%, respectively. Sequences corresponding to all 11 RVA genome segments were recovered. The assembled sequence lengths ranged from 711 nt (NSP5) to 3280 nt (VP1), and the predicted proteins ranged from 159 amino acids (NSP4) to 1081 amino acids (VP1). SD2503 was assigned the genotype constellation G3-P[59]-I3-R3-C3-M3-A9-N3-T3-E3-H6 (Table 1).

Segment-specific read-mapping analysis was performed to assess sequencing support for the 11 assembled RVA genome segments (Supplementary Table S2). Mean sequencing depths ranged from 93.18× for VP3 to 2652.05× for NSP3. Ten of the 11 segments showed 100.00% breadth of coverage at ≥1× depth across their assembled sequences. The remaining segment, NSP2, showed 95.29% breadth of coverage, with 52 nucleotide positions lacking mapped-read support. At the ≥10× depth threshold, coverage ranged from 91.14% for NSP5 to 99.82% for NSP3. These results provide quantitative read-mapping support for recovery of sequences representing all 11 RVA genome segments. However, complete coverage of the natural 5′ and 3′ termini was not independently demonstrated.

### 3.3. Divergent VP4 and Confirmation of P[59]

**Figure 2:**
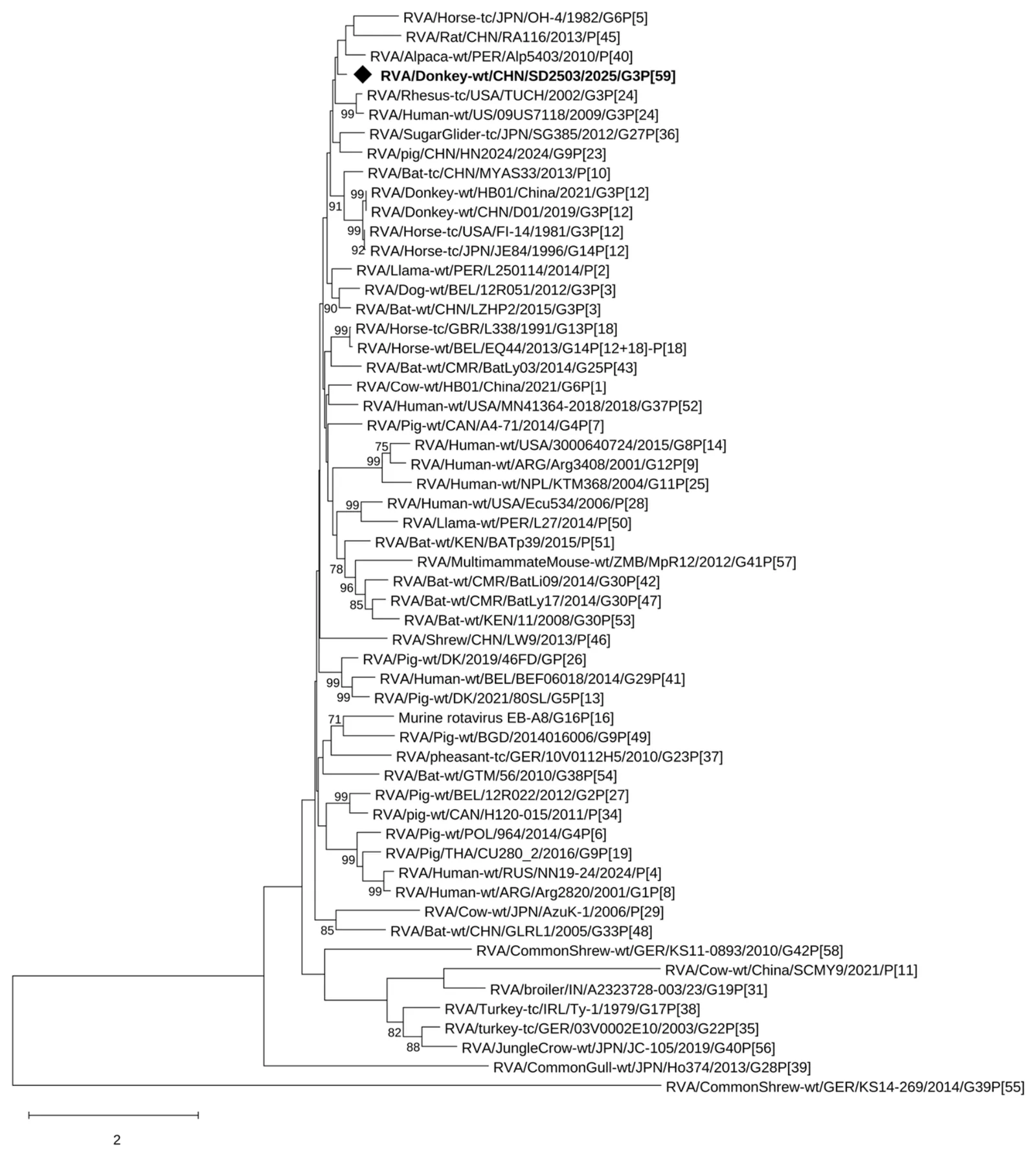
Maximum-likelihood phylogeny of *VP4*. Representative RVA P genotypes were included to place the RCWG-confirmed P[59] sequence in a broad genotype context. SD2503 is marked by a black diamond (◆). Bootstrap values ≥70% are shown. The tree was reconstructed in MEGA12 using the BIC-selected model and 1000 standard bootstrap replicates.

*VP4* was the most divergent segment of SD2503. Its closest hit in the available BLASTn analysis was the P[24] rhesus RVA strain TUCH (FJ816611.1), with 79.582% nucleotide identity across a 2346-nt alignment. This value was below the 80% nucleotide identity threshold used to assign *VP4* sequences to the same P genotype [4,5]. The *VP4* sequence was subsequently reviewed by the RCWG and confirmed as P[59]. In the broad *VP4* maximum-likelihood tree containing representatives of recognized P genotypes, SD2503 formed a distinct branch rather than clustering within the conventional equine P[12] group (Figure 2). Although the P[24] rhesus strain TUCH represented the closest match in the BLASTn analysis, the phylogenetic position of SD2503 in Figure 2 appeared closer to an alpaca-associated P[40] reference strain. This difference is not necessarily contradictory, because BLASTn nucleotide identity and maximum-likelihood phylogenetic placement reflect different analytical approaches, including differences in alignment coverage, reference sequence sampling, and evolutionary model-based inference.

### 3.4. VP7 Phylogeny Places SD2503 with an Atypical G3 Lineage

**Figure 3:**
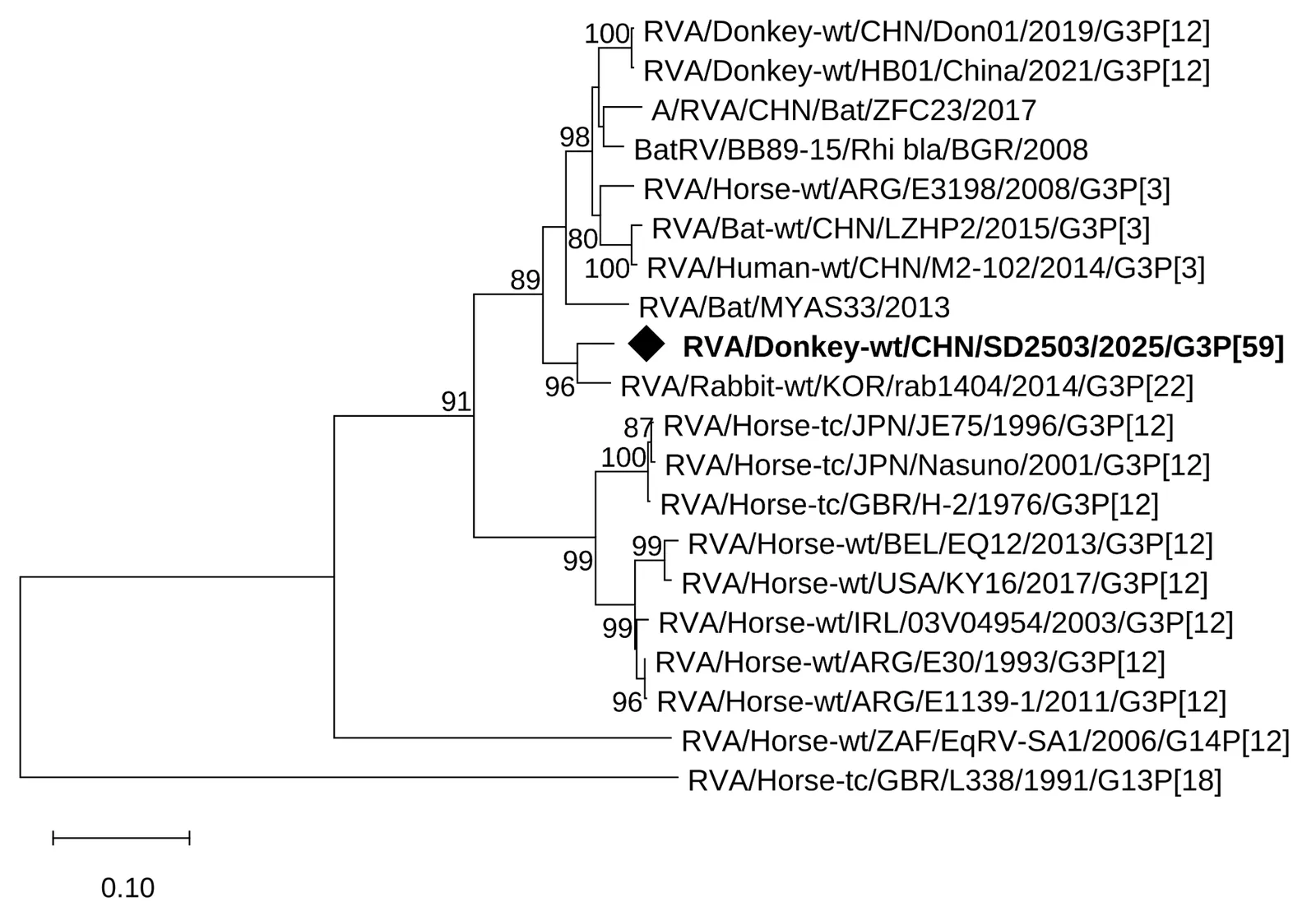
Maximum-likelihood phylogeny of *VP7*. SD2503 (◆) clustered with rabbit strain rab1404 with 96% bootstrap support. Bootstrap values ≥70% are shown. The tree was reconstructed in MEGA12 under the BIC-selected model with 1000 standard bootstrap replicates.

The *VP7* segment was assigned to G3 but did not group with the principal conventional equine G3P[12] cluster. Instead, SD2503 formed a sister relationship with rabbit strain rab1404, supported by a bootstrap value of 96% (Figure 3). This topology agreed with the BLASTn result, in which rab1404 was the closest *VP7* match at 95.45% nucleotide identity. Donkey strains Don01/2019 and HB01/2021 grouped elsewhere with bat-associated and atypical equine G3 viruses, emphasizing the diversity of G3 *VP7* lineages detected in equids.

### 3.5. Internal-Gene Phylogenies Reveal Topological Incongruence

**Figure 4:**
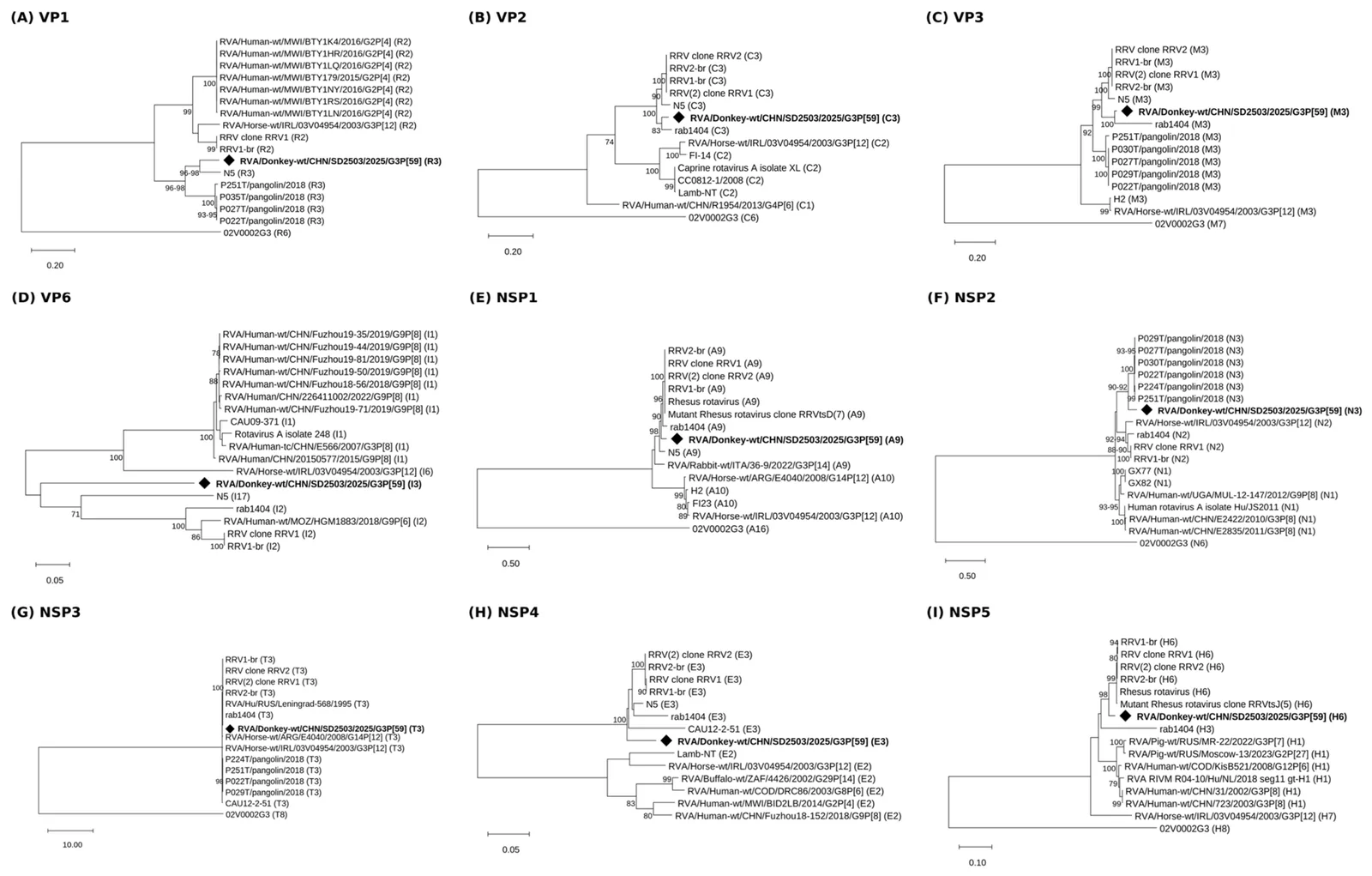
Maximum-likelihood phylogenies of the nine internal genome segments of SD2503: (**A**) *VP1*; (**B**) *VP2*; (**C**) *VP3*; (**D**) *VP6*; (**E**) *NSP1*; (**F**) *NSP2*; (**G**) *NSP3*; (**H**) *NSP4*; and (**I**) *NSP5*. SD2503 is indicated by a black diamond (◆). Bootstrap values ≥70% are shown. Each segment was analyzed independently in MEGA12 using its BIC-selected nucleotide substitution model and 1000 standard bootstrap replicates. Scale bars represent substitutions per site and differ among panels.

The nine internal-gene trees showed that SD2503 did not follow a single host-associated phylogenetic pattern (Figure 4). In the *VP1* (R3) phylogeny, SD2503 formed a sister relationship with rabbit strain N5 with 98% bootstrap support, within a broader lineage containing pangolin-associated R3 references. In the *VP2* (C3) phylogeny, SD2503 formed a sister relationship with rabbit strain rab1404 with 83% bootstrap support within a broader C3 lineage containing simian-associated references. Similarly, in the *VP3* (M3) phylogeny, SD2503 clustered with rab1404 with 100% bootstrap support within a broader M3 assemblage. *VP6* was assigned to I3 and remained distinct from the I1, I2, I6 and I17 reference groups represented in the selected dataset. *NSP1* (A9) fell within an A9 assemblage containing rabbit and rhesus/simian viruses. *NSP2* (N3) grouped with pangolin-associated N3 sequences, whereas *NSP3* (T3) was embedded within a broad T3 lineage containing simian, rabbit, equine and pangolin-associated viruses. *NSP4* was placed within E3, separate from the E2 cluster containing conventional equine strain 03V04954, and *NSP5* (H6) grouped near rhesus/simian H6 viruses. These segment-specific placements are incongruent with one another and with the conventional equine G3P[12] genomic background. Representative phylogenetically related reference strains identified from the nine internal-gene trees are summarized in Supplementary Table S3, together with their host origins, GenBank accession numbers, pairwise nucleotide identities with SD2503, and available bootstrap support values. These comparisons were interpreted as evidence of phylogenetic relationships rather than direct ancestry or interspecies transmission.

### 3.6. Genotype Constellation Indicates a Distinct Equid RVA Background

The complete constellation of SD2503 was G3-P[59]-I3-R3-C3-M3-A9-N3-T3-E3-H6. This differs markedly from the common equine G3P[12] constellation, typically G3-P[12]-I6-R2-C2-M3-A10-N2-T3-E2-H7 [13]. At the genotype level, SD2503 and the unusual equine strain E3198 (G3-P[3]-I3-R3-C3-M3-A9-N3-T3-E3-H6) share identical genotype assignments for ten genome segments, including *VP7*, *VP6*, *VP1*–*VP3*, and *NSP1*–*NSP5*, but differ in their *VP4* genotypes (P[59] versus P[3], respectively) [10]. This genotype-level similarity does not necessarily indicate close nucleotide sequence identity across all ten segments or establish a direct evolutionary relationship between the two strains. However, the individual SD2503 trees do not consistently place every segment immediately with E3198 or with any single reference strain. The genome is therefore best interpreted as belonging to a broader cross-species-associated RVA gene pool rather than as a direct derivative of one previously sampled virus.

## 4. Discussion

This study provides whole-genome characterization of an independently identified donkey RVA carrying the newly recognized P[59] genotype. Three observations are central to the interpretation of SD2503. First, *VP4* is sufficiently divergent from established P genotypes to cross the formal sequence boundary used in RVA classification, and its P[59] designation has been confirmed by the RCWG. Second, the nine internal genome segments have an unusual genotype constellation (I3-R3-C3-M3-A9-N3-T3-E3-H6), distinct from the internal-gene background commonly associated with equine G3P[12] viruses. Third, the 11 segment trees are topologically incongruent, linking SD2503 to different cross-species-associated RVA lineages depending on the segment examined. Together, these features indicate an unusual genomic background consistent with possible historical reassortment, although the specific evolutionary events remain unresolved.

The *VP4* finding is important because P-genotype diversity may be missed when surveillance is restricted to common equine G/P combinations. The 79.58% identity between SD2503 *VP4* and P[24] TUCH lies below the 80% same-genotype cutoff established in the whole-genome classification framework [4,5]. In the broad *VP4* tree, SD2503 remained distinct from P[12] equine viruses and from the P[24] reference cluster. The tree is not used here to redefine the genotype, because the formal P[59] assignment rests on RCWG confirmation; rather, it provides evolutionary context for the marked divergence of the sequence.

The *VP7* tree adds a second unusual feature. SD2503 grouped with rabbit strain rab1404 with 96% bootstrap support. Rab1404 itself has a mosaic G3-P[22]-I2-R3-C3-M3-A9-N2-T3-E3-H3 genome and was interpreted as the product of multiple reassortment events involving animal RVA lineages [26]. This relationship should not be read as evidence that rabbits directly transmitted the virus to donkeys. Rather, it indicates that closely related G3 genes have circulated in a wider mammalian RVA network that also includes bat-, simian-, feline/canine- and equine-associated viruses [13,27,28].

The internal genes reinforce this interpretation. Several SD2503 segments occupy genotype groups represented by simian RRV-like, rabbit, pangolin-associated or atypical equine viruses, whereas *VP6* is I3 and distinct from the I6 background of conventional equine G3P[12] strains. The overall genotype constellation is especially notable because it is identical to E3198 at ten genotype positions [10]. E3198 and Indian bat-associated equine G3P[3] strains illustrate that equids can harbor RVA genomes assembled from lineages that are not typical of the dominant equine G3P[12]/G14P[12] population [10,13]. Nevertheless, genotype identity does not establish direct ancestry. The absence of consistent nearest-neighbor relationships across the SD2503 trees argues against describing SD2503 simply as an E3198 derivative with a replaced *VP4* segment.

Donkey RVA data remain sparse. Don01/2019 demonstrated that severe enteritis in donkey foals can be associated with a G3P[12] virus whose outer-capsid genes have discordant evolutionary affinities [14]. HB01/2021 further linked donkey G3 viruses with atypical equine/bat-related *VP7* diversity [15]. The recent report of HB2503 as another G3P[59] donkey RVA is epidemiologically noteworthy [16], but direct genomic comparison is currently limited by the lack of publicly available nucleotide sequences in that report. Consequently, the present data support independent detection of P[59] in a Shandong donkey RVA but do not establish the geographic origin, direction of spread, or prevalence of this genotype.

Reassortment and interspecies transmission are well documented in RVA evolution [2,6,27,28,29,30,31]. Simian RRV and TUCH themselves have complex genome constellations [27], and feline/canine-like, bat, rabbit and equine RVAs provide examples in which individual segments trace to different host-associated lineages [13,14,27,28,29,31]. SD2503 fits this broader evolutionary pattern. Its unusual genotype constellation and incongruent segment-specific phylogenetic relationships are consistent with possible historical reassortment involving cross-species-associated RVA lineages. However, the available data do not establish the number, timing, or direction of such events, and identifying specific parental viruses or transmission sources would require denser contemporaneous sampling and additional complete genomes from potentially relevant animal hosts.

This study has several limitations. First, only one SD2503 isolate was characterized from a diarrheic donkey foal. Although the virus was recovered and characterized, its detection in a single clinical case does not establish a causal relationship with diarrhea. Additional clinical investigations, epidemiological studies, and experimental evidence would be required to determine the prevalence and pathogenic significance of P[59] RVA in donkeys. Second, available RVA reference sequences are unevenly distributed across host species and geographic regions, and some potentially relevant animal RVA lineages remain insufficiently sampled or are represented only by partial sequences. Consequently, phylogenetic relationships between SD2503 and RVAs associated with other mammalian hosts cannot establish direct interspecies transmission, identify the donor host, or determine the direction or timing of transmission and reassortment events. Third, although sequences corresponding to all 11 RVA genome segments were recovered, the complete 5′ and 3′ genomic termini were not independently verified, and read-mapping analysis identified incomplete coverage of the assembled *NSP2* sequence. These limitations should be considered when interpreting the genomic and evolutionary findings. Nevertheless, the combination of RCWG-confirmed *VP4* genotyping and 11-segment phylogenetic analysis provides a useful framework for recognizing SD2503 as a genetically unusual donkey RVA.

## 5. Conclusions

SD2503 is an independently identified donkey RVA with the genotype constellation G3-P[59]-I3-R3-C3-M3-A9-N3-T3-E3-H6. Its *VP4* gene belongs to the newly recognized P[59] genotype, while its remaining genome segments show discordant phylogenetic relationships with several atypical mammalian RVA lineages. These findings reveal an unusual genomic background and are consistent with possible historical reassortment. However, the number, timing, direction, and parental lineages of the potential reassortment events cannot be determined from the present data. Broader genomic and epidemiological surveillance of RVA in donkeys, horses, wildlife, and other sympatric mammals, together with additional clinical investigations, is needed to determine the prevalence, host distribution, clinical significance, and evolutionary history of P[59] RVA strains.

## Figures and Tables

**Table 1:** Genome segment characteristics of SD2503 and their closest BLASTn matches.

Segment	Genotype	Length (nt)	Protein (aa)	Closest Match	Accession	Identity (%)
VP1	R3	3280	1081	2012841174/2012, human G8P[14]	KJ411432	93.415
VP2	C3	2703	887	MS2015-1-0001, human G3P[10]	LC576609	96.707
VP3	M3	2583	844	HB01/China/2021, donkey G3P[12]	ON377049	94.949
VP4	P[59]	2355	776	TUCH/2002, rhesus G3P[24]	FJ816611	79.582
VP6	I3	1413	391	IAL-R566/2011, human G12P[9]	PP025572	89.788
VP7	G3	1055	297	rab1404/2014, rabbit G3P[22]	MK751437	95.450
NSP1	A9	1624	492	Rotavirus A type 1 NSP1	OP963640	95.722
NSP2	N3	1105	288	E3198/2008, horse G3P[3]	JX036372	93.732
NSP3	T3	1132	310	RRV/Rotashield, simian	HQ846849	95.005
NSP4	E3	788	159	K8/1977, human G1P[9]	JQ713651	95.393
NSP5	H6	711	198	E3198/2008, horse G3P[3]	JX036375	98.628

## Data Availability

The nucleotide sequences of the 11 genome segments of RVA/Donkey-wt/CHN/SD2503/2025/G3P[59] generated in this study have been deposited in GenBank under accession numbers QB141057–QB141067.

## Supplementary Materials

The following supporting information can be downloaded at: https://www.mdpi.com/article/10.3390/ani16213511/s1, Table S1. Reference rotavirus A strains used for phylogenetic analyses of the 11 genome segments, including strain designations, GenBank accession numbers, host species, and segment genotypes. Table S2. Segment-specific read-mapping depth and breadth of coverage for the 11 assembled genome segments of rotavirus A strain SD2503. Table S3. Representative phylogenetically related rotavirus A strains for the nine internal genome segments of SD2503: nucleotide identities and node-specific bootstrap support.
