# Supplementary material for: Whole-Genome Characterization of a G3P[59] Rotavirus A Strain from a Diarrheic Donkey Foal Reveals an Unusual Genomic Background
Source: Animals (Basel). 2026 Nov 6;16(21):3511. doi: 10.3390/ani16213511 (PMC13761555; doi:10.3390/ani16213511)
Supplement: Supplementary file 1 [file animals-16-03511-s001.zip › animals-4645106-supplementary.pdf]

Table S1. Reference rotavirus A strains used for phylogenetic analyses of the 11 genome segments, including strain designations, GenBank accession numbers, host species, and segment genotypes.

Genome segment	Reference strain designation	GenBank accession number	Host species	Segment genotype
VP4	RVA/Donkey-wt/CHN/SD2503/2025/G3P[59]	QB141060	Donkey	P[59]
VP4	RVA/Donkey-wt/HB01/China/2021/G3P[12]	ON377050	Donkey	P[12]
VP4	RVA/Pig-wt/DK/2019/46FD/GP[26]	PV891421	Pig	P[26]
VP4	RVA/Human-wt/USA/Ecu534/2006/P[28]	EU805773	Human	P[28]
VP4	RVA/Cow-wt/JPN/AzuK-1/2006/P[29]	AB454420	Cattle	P[29]
VP4	RVA/Rhesus-tc/USA/TUCH/2002/G3P[24]	FJ816611	Rhesus macaque	P[24]
VP4	RVA/Human-wt/NPL/KTM368/2004/G11P[25]	GU199495	Human	P[25]
VP4	RVA/Horse-tc/GBR/L338/1991/G13P[18]	JF712558	Horse	P[18]
VP4	RVA/turkey-tc/GER/03V0002E10/2003/G22P[35]	JX204825	Turkey	P[35]
VP4	RVA/pheasant-tc/GER/10V0112H5/2010/G23P[37]	JX204814	Pheasant	P[37]
VP4	RVA/Horse-tc/JPN/OH-4/1982/G6P[5]	KC815661	Horse	P[5]
VP4	Murine_rotavirus_EB-A8/G16P[16]	KJ477108	Mouse	P[16]
VP4	RVA/Dog-wt/BEL/12R051/2012/G3P[3]	KF614069	Dog	P[3]
VP4	RVA/Human-wt/US/09US7118/2009/G3P[24]	KF541284	Human	P[24]
VP4	RVA/Pig-wt/BEL/12R022/2012/G2P[27]	KM820721	Pig	P[27]
VP4	RVA/SugarGlider-tc/JPN/SG385/2012/G27P[36]	AB971763	Sugar glider	P[36]
VP4	RVA/Horse-tc/USA/FI-14/1981/G3P[12]	KM454484	Horse	P[12]
VP4	RVA/Horse-wt/BEL/EQ44/2013/G14P[12+18]-P[18]	KM515869	Horse	P[12]
VP4	RVA/Horse-tc/JPN/JE84/1996/G14P[12]	AB908926	Horse	P[12]
VP4	RVA/Turkey-tc/IRL/Ty-1/1979/G17P[38]	LC088110	Turkey	P[38]
VP4	RVA/CommonGull-wt/JPN/Ho374/2013/G28P[39]	LC088221	Common gull	P[39]
VP4	RVA/Shrew/CHN/LW9/2013/P[46]	KU243621	Shrew	P[46]

Genome segment	Reference strain designation	GenBank accession number	Host species	Segment genotype
VP4	RVA/Rat/CHN/RA116/2013/P[45]	KU243630	Rat	P[45]
VP4	RVA/Human-wt/BEL/BEF06018/2014/G29P[41]	KU128895	Human	P[41]
VP4	RVA/Bat-wt/CMR/BatLi09/2014/G30P[42]	KX268757	Bat	P[42]
VP4	RVA/Bat-wt/CMR/BatLy03/2014/G25P[43]	KX268779	Bat	P[43]
VP4	RVA/Bat-wt/CMR/BatLy17/2014/G30P[47]	KX268790	Bat	P[47]
VP4	RVA/pig-wt/CAN/H120-015/2011/P[34]	KU954437	Pig	P[34]
VP4	RVA/Bat-wt/CHN/GLRL1/2005/G33P[48]	KX814933	Bat	P[48]
VP4	RVA/Bat-wt/CHN/LZHP2/2015/G3P[3]	KX814943	Bat	P[3]
VP4	RVA/Alpaca-wt/PER/Alp5403/2010/P[40]	KY971956	Alpaca	P[40]
VP4	RVA/Llama-wt/PER/L27/2014/P[50]	KY972021	Llama	P[50]
VP4	RVA/Llama-wt/PER/L250114/2014/P[2]	KY972025	Llama	P[2]
VP4	RVA/Pig-wt/BGD/2014016006/G9P[49]	KY905314	Pig	P[49]
VP4	RVA/Pig-wt/CAN/A4-71/2014/G4P[7]	KY909866	Pig	P[7]
VP4	RVA/Pig/THA/CU280_2/2016/G9P[19]	MH428380	Pig	P[19]
VP4	RVA/Bat-wt/KEN/BATp39/2015/P[51]	MH285840	Bat	P[51]
VP4	RVA/Donkey-wt/CHN/D01/2019/G3P[12]	MT211950	Donkey	P[12]
VP4	RVA/JungleCrow-wt/JPN/JC-105/2019/G40P[56]	LC634425	Jungle crow	P[56]
VP4	RVA/MultimammateMouse-wt/ZMB/MpR12/2012/G41P[57]	LC638701	Mouse	P[57]
VP4	RVA/CommonShrew-wt/GER/KS14-269/2014/G39P[55]	MZ054767	Shrew	P[55]
VP4	RVA/Cow-wt/HB01/China/2021/G6P[1]	OM643802	Cattle	P[1]
VP4	RVA/Human-wt/USA/MN41364-2018/2018/G37P[52]	MW752841	Human	P[52]
VP4	RVA/Cow-wt/China/SCMY9/2021/P[11]	ON012989	Cattle	P[11]
VP4	RVA/CommonShrew-wt/GER/KS11-0893/2010/G42P[58]	ON988156	Shrew	P[58]
VP4	RVA/Human-wt/USA/3000640724/2015/G8P[14]	ON737299	Human	P[14]
VP4	RVA/Pig-wt/POL/964/2014/G4P[6]	OQ982430	Pig	P[6]

Genome segment	Reference strain designation	GenBank accession number	Host species	Segment genotype
VP4	RVA/Bat-wt/KEN/11/2008/G30P[53]	OQ190907	Bat	P[53]
VP4	RVA/Bat-wt/GTM/56/2010/G38P[54]	OQ190918	Bat	P[54]
VP4	RVA/Human-wt/RUS/NN19-24/2024/P[4]	PQ376909	Human	P[4]
VP4	RVA/Pig-wt/DK/2021/80SL/G5P[13]	PV891398	Pig	P[13]
VP4	RVA/broiler/IN/A2323728-003/23/G19P[31]	PX115335	Broiler chicken	P[31]
VP4	RVA/Human-wt/ARG/Arg2820/2001/G1P[8]	PV769842	Human	P[8]
VP4	RVA/Human-wt/ARG/Arg3408/2001/G12P[9]	PV769848	Human	P[9]
VP4	RVA/pig/CHN/HN2024/2024/G9P[23]	PZ488420	Pig	P[23]
VP4	RVA/Bat-tc/CHN/MYAS33/2013/P[10]	KF649187	Bat	P[10]
VP7	RVA/Donkey-wt/CHN/SD2503/2025/G3P_59	QB141065	Donkey	G3
VP7	RVA/Donkey-wt/CHN/Don01/2019/G3P_12	MT211949	Donkey	G3
VP7	RVA/Donkey-wt/HB01/China/2021/G3P_12	ON377052	Donkey	G3
VP7	RVA/Rabbit-wt/KOR/Rab1404/2014/G3P[22]	MK751437	Rabbit	G3
VP7	A/RVA/CHN/Bat/ZFC23/2017	MK292706	Bat	G3
VP7	RVA/Bat/MYAS33/2013	KF649188	Bat	G3
VP7	BatRV/BB89-15/Rhi_bla/BGR/2008	KM214428	Bat	G3
VP7	RVA/Bat-wt/CHN/LZHP2/2015/G3P_3	KX814942	Bat	G3
VP7	RVA/Human-wt/CHN/M2-102/2014/G3P_3	KU597744	Human	G3
VP7	RVA/Horse-wt/ARG/E3198/2008/G3P_3	JX036370	Horse	G3
VP7	RVA/Horse-tc/GBR/H-2/1976/G3P_12	KM454497	Horse	G3
VP7	RVA/Horse-tc/JPN/JE75/1996/G3P_12	AB046466	Horse	G3
VP7	RVA/Horse-tc/JPN/Nasuno/2001/G3P_12	AB197846	Horse	G3
VP7	RVA/Horse-wt/BEL/EQ12/2013/G3P_12	KM515874	Horse	G3
VP7	RVA/Horse-wt/ARG/E30/1993/G3P_12	JF712571	Horse	G3
VP7	RVA/Horse-wt/ARG/E1139-1/2011/G3P_12	KP116037	Horse	G3

Genome segment	Reference strain designation	GenBank accession number	Host species	Segment genotype
VP7	RVA/Horse-wt/USA/KY16/2017/G3P_12	MG970175	Horse	G3
VP7	RVA/Horse-wt/IRL/03V04954/2003/G3P_12	JN903518	Horse	G3
VP7	RVA/Horse-wt/ZAF/EqRV-SA1/2006/G14P_12	JQ345496	Horse	G14
VP7	RVA/Horse-tc/GBR/L338/1991/G13P_18	JF712560	Horse	G13
VP1	RVA/Donkey-wt/CHN/SD2503/2025/G3P[59] (R3)	QB141057	Donkey	R3
VP1	N5 (R3)	JQ423902	Rabbit	R3
VP1	P027T/pangolin/2018 (R3)	ON059857	Pangolin	R3
VP1	P022T/pangolin/2018 (R3)	ON059856	Pangolin	R3
VP1	P035T/pangolin/2018 (R3)	ON059858	Pangolin	R3
VP1	P251T/pangolin/2018 (R3)	ON059859	Pangolin	R3
VP1	RVA/Human-wt/MWI/BTY1NY/2016/G2P[4] (R2)	OP717168	Human	R2
VP1	RVA/Human-wt/MWI/BTY1RS/2016/G2P[4] (R2)	OP717170	Human	R2
VP1	RVA/Human-wt/MWI/BTY1LN/2016/G2P[4] (R2)	OP717164	Human	R2
VP1	RVA/Human-wt/MWI/BTY1K4/2016/G2P[4] (R2)	OP717161	Human	R2
VP1	RVA/Human-wt/MWI/BTY179/2015/G2P[4] (R2)	OP717151	Human	R2
VP1	RVA/Human-wt/MWI/BTY1HR/2016/G2P[4] (R2)	OP717159	Human	R2
VP1	RVA/Human-wt/MWI/BTY1LQ/2016/G2P[4] (R2)	OP717165	Human	R2
VP1	RVA/Horse-wt/IRL/03V04954/2003/G3P[12] (R2)	JN903528	Horse	R2
VP1	RRV clone RRV1 (R2)	MT226612	Rhesus macaque	R2
VP1	RRV1-br (R2)	MT226623	Rhesus macaque	R2
VP1	02V0002G3 (R6)	FJ169853	Chicken	R6
VP2	RVA/Donkey-wt/CHN/SD2503/2025/G3P[59] (C3)	QB141058	Donkey	C3
VP2	rab1404 (C3)	MK751433	Rabbit	C3
VP2	RRV clone RRV1 (C3)	MT226613	Rhesus macaque	C3
VP2	RRV clone RRV2 (C3)	MT226635	Rhesus macaque	C3

Genome segment	Reference strain designation	GenBank accession number	Host species	Segment genotype
VP2	RRV1-br (C3)	MT226624	Rhesus macaque	C3
VP2	RRV2-br (C3)	MT226646	Rhesus macaque	C3
VP2	N5 (C3)	JQ423903	Rabbit	C3
VP2	Caprine rotavirus A isolate XL (C2)	JQ004971	Goat	C2
VP2	CC0812-1/2008 (C2)	HQ834198	Goat	C2
VP2	RVA/Horse-wt/IRL/03V04954/2003/G3P[12] (C2)	JN903526	Horse	C2
VP2	Lamb-NT (C2)	FJ031025	Sheep	C2
VP2	RVA/Human-wt/CHN/R1954/2013/G4P[6] (C1)	KF726070	Human	C1
VP2	FI-14 (C2)	KM454482	Horse	C2
VP2	02V0002G3 (C6)	FJ169854	Chicken	C6
VP3	RVA/Donkey-wt/CHN/SD2503/2025/G3P[59] (M3)	QB141059	Donkey	M3
VP3	rab1404 (M3)	MK751434	Rabbit	M3
VP3	RRV clone RRV1 (M3)	MT226614	Rhesus macaque	M3
VP3	RRV clone RRV2 (M3)	MT226636	Rhesus macaque	M3
VP3	RRV1-br (M3)	MT226625	Rhesus macaque	M3
VP3	RRV2-br (M3)	MT226647	Rhesus macaque	M3
VP3	N5 (M3)	JQ423904	Rabbit	M3
VP3	P030T/pangolin/2018 (M3)	ON059866	Pangolin	M3
VP3	P027T/pangolin/2018 (M3)	ON059864	Pangolin	M3
VP3	P029T/pangolin/2018 (M3)	ON059865	Pangolin	M3
VP3	P022T/pangolin/2018 (M3)	ON059863	Pangolin	M3
VP3	P251T/pangolin/2018 (M3)	ON059867	Pangolin	M3
VP3	H2 (M3)	KM454494	Horse	M3
VP3	RVA/Horse-wt/IRL/03V04954/2003/G3P[12] (M3)	JN903524	Horse	M3
VP3	02V0002G3 (M7)	FJ169855	Chicken	M7

Genome segment	Reference strain designation	GenBank accession number	Host species	Segment genotype
VP6	RVA/Donkey-wt/CHN/SD2503/2025/G3P[59] (I3)	QB141062	Donkey	I3
VP6	RVA/Human/CHN/20150577/2015/G9P[8] (I1)	OR453311	Human	I1
VP6	Rotavirus A isolate 248 (I1)	OP950163	Not specified	I1
VP6	CAU09-371 (I1)	JF766593	Human	I1
VP6	RVA/Human-tc/CHN/E566/2007/G3P[8] (I1)	KF371671	Human	I1
VP6	RVA/Human-wt/CHN/Fuzhou19-35/2019/G9P[8] (I1)	MW384312	Human	I1
VP6	RVA/Human-wt/CHN/Fuzhou19-44/2019/G9P[8] (I1)	MW384356	Human	I1
VP6	RVA/Human-wt/CHN/Fuzhou19-50/2019/G9P[8] (I1)	MW384378	Human	I1
VP6	RVA/Human/CHN/226411002/2022/G9P[8] (I1)	OR453305	Human	I1
VP6	RVA/Human-wt/CHN/Fuzhou19-81/2019/G9P[8] (I1)	MW384279	Human	I1
VP6	RVA/Human-wt/CHN/Fuzhou18-56/2018/G9P[8] (I1)	MW384180	Human	I1
VP6	RVA/Human-wt/CHN/Fuzhou19-71/2019/G9P[8] (I1)	MW384268	Human	I1
VP6	RVA/Human-wt/MOZ/HGM1883/2018/G9P[6] (I2)	PP848588	Human	I2
VP6	RVA/Horse-wt/IRL/03V04954/2003/G3P[12] (I6)	JN903520	Horse	I6
VP6	RRV clone RRV1 (I2)	MT226617	Rhesus macaque	I2
VP6	RRV1-br (I2)	MT226628	Rhesus macaque	I2
VP6	rab1404 (I2)	MK751436	Rabbit	I2
VP6	N5 (I17)	JQ423906	Rabbit	I17
NSP1	RVA/Donkey-wt/CHN/SD2503/2025/G3P[59] (A9)	QB141061	Donkey	A9
NSP1	RRV1-br (A9)	MT226627	Rhesus macaque	A9
NSP1	RRV clone RRV2 (A9)	MT226638	Rhesus macaque	A9
NSP1	RRV2-br (A9)	MT226649	Rhesus macaque	A9
NSP1	RRV clone RRV1 (A9)	MT226616	Rhesus macaque	A9
NSP1	Rhesus rotavirus (A9)	HQ665465	Rhesus macaque	A9
NSP1	Mutant Rhesus rotavirus clone RRVtsD(7) (A9)	HQ665462	Rhesus macaque	A9

Genome segment	Reference strain designation	GenBank accession number	Host species	Segment genotype
NSP1	rab1404 (A9)	MK751428	Rabbit	A9
NSP1	N5 (A9)	JQ423897	Rabbit	A9
NSP1	RVA/Rabbit-wt/ITA/36-9/2022/G3[P14] (A9)	PQ822048	Rabbit	A9
NSP1	RVA/Horse-wt/ARG/E4040/2008/G14P[12] (A10)	JN872871	Horse	A10
NSP1	FI23 (A10)	KM454509	Horse	A10
NSP1	H2 (A10)	KM454498	Horse	A10
NSP1	RVA/Horse-wt/IRL/03V04954/2003/G3P[12] (A10)	JN903516	Horse	A10
NSP1	02V0002G3 (A16)	FJ169857	Chicken	A16
NSP2	RVA/Donkey-wt/CHN/SD2503/2025/G3P[59] (N3)	QB141064	Donkey	N3
NSP2	P224T/pangolin/2018 (N3)	ON059888	Pangolin	N3
NSP2	P022T/pangolin/2018 (N3)	ON059884	Pangolin	N3
NSP2	P029T/pangolin/2018 (N3)	ON059886	Pangolin	N3
NSP2	P027T/pangolin/2018 (N3)	ON059885	Pangolin	N3
NSP2	P251T/pangolin/2018 (N3)	ON059889	Pangolin	N3
NSP2	P030T/pangolin/2018 (N3)	ON059887	Pangolin	N3
NSP2	GX77 (N1)	KF447846	Human	N1
NSP2	GX82 (N1)	KF447868	Human	N1
NSP2	RVA/Human-wt/CHN/E2422/2010/G3P[8] (N1)	KF371836	Human	N1
NSP2	RVA/Human-wt/UGA/MUL-12-147/2012/G9P[8] (N1)	KX632305	Human	N1
NSP2	Human rotavirus A isolate Hu/JS2011 (N1)	MF580904	Human	N1
NSP2	RVA/Human-wt/CHN/E2835/2011/G3P[8] (N1)	KF371869	Human	N1
NSP2	RVA/Horse-wt/IRL/03V04954/2003/G3P[12] (N2)	JN903514	Horse	N2
NSP2	RRV clone RRV1 (N2)	MT226619	Rhesus macaque	N2
NSP2	RRV1-br (N2)	MT226630	Rhesus macaque	N2
NSP2	rab1404 (N2)	MK751429	Rabbit	N2

Genome segment	Reference strain designation	GenBank accession number	Host species	Segment genotype
NSP2	02V0002G3 (N6)	FJ169860	Chicken	N6
NSP3	RVA/Donkey-wt/CHN/SD2503/2025/G3P[59] (T3)	QB141063	Donkey	T3
NSP3	RRV2-br (T3)	MT226651	Rhesus macaque	T3
NSP3	RRV clone RRV1 (T3)	MT226618	Rhesus macaque	T3
NSP3	RRV1-br (T3)	MT226629	Rhesus macaque	T3
NSP3	RRV clone RRV2 (T3)	MT226640	Rhesus macaque	T3
NSP3	RVA/Hu/RUS/Leningrad-568/1995 (T3)	OP950159	Human	T3
NSP3	rab1404 (T3)	MK751430	Rabbit	T3
NSP3	RVA/Horse-wt/ARG/E4040/2008/G14P[12] (T3)	JN872873	Horse	T3
NSP3	P224T/pangolin/2018 (T3)	ON059882	Pangolin	T3
NSP3	P251T/pangolin/2018 (T3)	ON059883	Pangolin	T3
NSP3	CAU12-2-51 (T3)	KJ187596	Human	T3
NSP3	P022T/pangolin/2018 (T3)	ON059879	Pangolin	T3
NSP3	P029T/pangolin/2018 (T3)	ON059881	Pangolin	T3
NSP3	RVA/Horse-wt/IRL/03V04954/2003/G3P[12] (T3)	JN903512	Horse	T3
NSP3	02V0002G3 (T8)	FJ169859	Chicken	T8
NSP4	RVA/Donkey-wt/CHN/SD2503/2025/G3P[59] (E3)	QB141066	Donkey	E3
NSP4	RRV clone RRV2 (E3)	MT226643	Rhesus macaque	E3
NSP4	RRV2-br (E3)	MT226654	Rhesus macaque	E3
NSP4	N5 (E3)	JQ423900	Rabbit	E3
NSP4	RRV clone RRV1 (E3)	MT226621	Rhesus macaque	E3
NSP4	RRV1-br (E3)	MT226632	Rhesus macaque	E3
NSP4	rab1404 (E3)	MK751431	Rabbit	E3
NSP4	CAU12-2-51 (E3)	KJ187597	Human	E3
NSP4	RVA/Buffalo-wt/ZAF/4426/2002/G29P[14] (E2)	MT234367	Water buffalo	E2

Genome segment	Reference strain designation	GenBank accession number	Host species	Segment genotype
NSP4	RVA/Human-wt/COD/DRC86/2003/G8P[6] (E2)	DQ005116	Human	E2
NSP4	Lamb-NT (E2)	FJ031022	Sheep	E2
NSP4	RVA/Human-wt/MWI/BID2LB/2014/G2P[4] (E2)	OP718029	Human	E2
NSP4	RVA/Human-wt/CHN/Fuzhou18-152/2018/G9P[8] (E2)	MW384108	Human	E2
NSP4	RVA/Horse-wt/IRL/03V04954/2003/G3P[12] (E2)	JN903510	Horse	E2
NSP5	RVA/Donkey-wt/CHN/SD2503/2025/G3P[59] (H6)	QB141067	Donkey	H6
NSP5	Rhesus rotavirus (H6)	HQ665467	Rhesus macaque	H6
NSP5	RRV clone RRV2 (H6)	MT226644	Rhesus macaque	H6
NSP5	Mutant Rhesus rotavirus clone RRVtsJ(5) (H6)	HQ665464	Rhesus macaque	H6
NSP5	RRV1-br (H6)	MT226633	Rhesus macaque	H6
NSP5	RRV clone RRV1 (H6)	MT226622	Rhesus macaque	H6
NSP5	RRV2-br (H6)	MT226655	Rhesus macaque	H6
NSP5	RVA/Pig-wt/RUS/MR-22/2022/G3P[7] (H1)	PP874436	Pig	H1
NSP5	RVA/Pig-wt/RUS/Moscow-13/2023/G2P[27] (H1)	PP874447	Pig	H1
NSP5	RVA_RIVM_R04-10/Hu/NL/2018_seg11_gt-H1 (H1)	OP255988	Human	H1
NSP5	RVA/Human-wt/CHN/31/2002/G3P[8] (H1)	KF371707	Human	H1
NSP5	RVA/Human-wt/CHN/723/2003/G3P[8] (H1)	KF371718	Human	H1
NSP5	RVA/Human-wt/COD/KisB521/2008/G12P[6] (H1)	KJ870921	Human	H1
NSP5	RVA/Horse-wt/IRL/03V04954/2003/G3P[12] (H7)	JN903508	Horse	H7
NSP5	rab1404 (H3)	MK751432	Rabbit	H3
NSP5	02V0002G3 (H8)	FJ169863	Chicken	H8

Note: The study strain SD2503 is included for completeness. Segment genotypes are reported according to the corresponding RVA genome segment.

Table S2. Segment-specific read-mapping depth and breadth of coverage for the 11 assembled genome segments of rotavirus A strain SD2503.

Segment	Assembly length (nt)	Mean depth (×)	Median depth (×)	Coverage ≥1× (%)	Coverage ≥10× (%)	Coverage ≥100× (%)	Zero-depth positions	Minimum depth (×)	Maximum depth (×)
VP1	3280	106.14	107.00	100.00	97.53	60.24	0	2	189
VP2	2703	193.95	131.00	100.00	99.48	72.40	0	1	787
VP3	2583	93.18	91.00	100.00	98.88	44.75	0	1	207
VP4	2355	143.80	141.00	100.00	99.66	86.58	0	2	253
VP6	1413	745.91	784.00	100.00	93.49	87.69	0	1	1,647
VP7	1055	333.84	335.00	100.00	99.72	94.03	0	3	703
NSP1	1624	669.79	539.50	100.00	96.31	95.63	0	1	1,992
NSP2	1105	776.61	789.00	95.29	94.30	92.04	52	0	1,291
NSP3	1132	2,652.05	1,423.00	100.00	99.82	95.58	0	1	27,031
NSP4	788	778.54	523.00	100.00	92.77	91.75	0	4	1,696
NSP5	711	969.41	885.00	100.00	91.14	87.48	0	1	1,872

Note 1. Coverage breadth (%) is the proportion of nucleotide positions in each assembled SD2503 reference sequence with mapped-read depth ≥1×, ≥10×, or ≥100×. Mean and median depth include zero-depth positions.

Note 2. Paired-end reads were aligned against the 11 assembled SD2503 segment sequences using BWA-MEM (bwa mem -t 4); the sorted alignment was processed using SAMtools (sort, index, flagstat, idxstats and depth -aa). The script accepts external FASTQ inputs and does not execute fastp; the preprocessing status of the supplied reads cannot be established from the script alone.

Note 3. Ten assembled segments had 100.00% coverage at ≥1×, whereas NSP2 had 95.29% coverage with 52 zero-depth positions. These are reference-based mapping statistics and do not independently confirm the accuracy of every assembled nucleotide or the complete natural 5′ and 3′ genome termini.

Note 4. Data source: SD2503_11_segment_coverage.csv; analysis script: SD2503_run_coverage(1).sh. Values are reported to two decimal places where appropriate.

Table S3. Representative phylogenetically related rotavirus A strains for the nine internal genome segments of SD2503: nucleotide identities and node-specific bootstrap support.

Segment	Genotype	Reference strain	GenBank accession	Host	Nt identity (%)	Compared nt	Bootstrap (%)	Supported node / interpretation
VP1	R3	N5	JQ423902	Rabbit	87.08	3275	98	Supported sister pair: SD2503 + N5
VP2	C3	rab1404	MK751433	Rabbit	93.87	2661	83	Supported sister pair: SD2503 + rab1404
VP3	M3	rab1404	MK751434	Rabbit	94.3	1823	100	Supported sister pair: SD2503 + rab1404
VP6	I3	RVA/Human-wt/MOZ/HGM1883/2018/G9P[6]	PP848588	Human	80.01	1356	100	Broader clade (6 taxa) including SD2503 and HGM1883; not a sister pair
NSP1	A9	RRV1-br	MT226627	Simian (rhesus)	91.3	1586	91	Broader clade (8 taxa) including SD2503 and RRV1-br; not a sister pair
NSP2	N3	P022T/pangolin/2018	ON059884	Pangolin-associated	86.91	1047	91	Broader clade (7 taxa) including SD2503 and P022T; not a sister pair
NSP3	T3	RRV1-br	MT226629	Simian (rhesus)	94.57	1068	100	Broader clade (7 taxa) including SD2503 and RRV1-br; not a sister pair
NSP4	E3	N5	JQ423900	Rabbit	94.49	744	NR	No uniquely attributable supported SD2503–N5 node in original ML Newick
NSP5	H6	Rhesus rotavirus	HQ665467	Simian (rhesus)	95.47	662	99	Broader clade (7 taxa) including SD2503 and rhesus RVA; not a sister pair

Methods: Nucleotide identity values and comparable-site counts were retained from pairwise comparisons against final segment-specific FASTA datasets; these are not the nearest BLASTn matches presented in manuscript Table 1. Bootstrap values were independently verified using the original MEGA12 maximum-likelihood Newick exports (1,000 standard bootstrap replicates) and rounded to whole percentages.

Bootstrap interpretation: VP1 (N5), VP2 (rab1404), and VP3 (rab1404) describe supported two-taxon sister pairs. Values shown for VP6 (six-taxon clade), NSP1 (eight-taxon clade), NSP2 (seven-taxon clade), NSP3 (seven-taxon clade), and NSP5 (seven-taxon clade) represent support for a larger clade containing SD2503 and the reference strain—not a uniquely supported sister relationship. The supported relationship to N5 for NSP4 could not be resolved (NR).

Important caveat: A reference strain is a representative of a phylogenetically relevant lineage and is not necessarily the uniquely closest relative or direct ancestral/donor strain. Phylogenetic proximity alone does not indicate interspecies transmission direction. For VP3, the reported 94.30% identity is based on 1,823 comparable nucleotide positions, not a full-length VP3 comparison.
